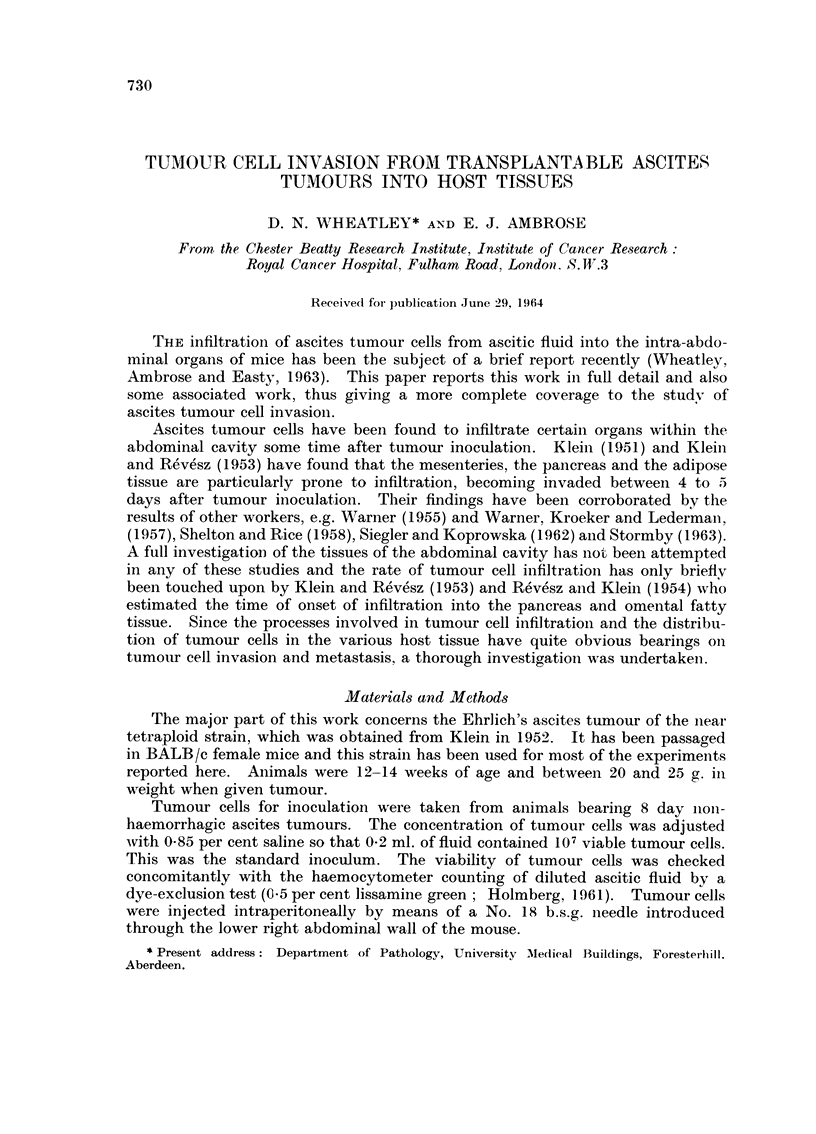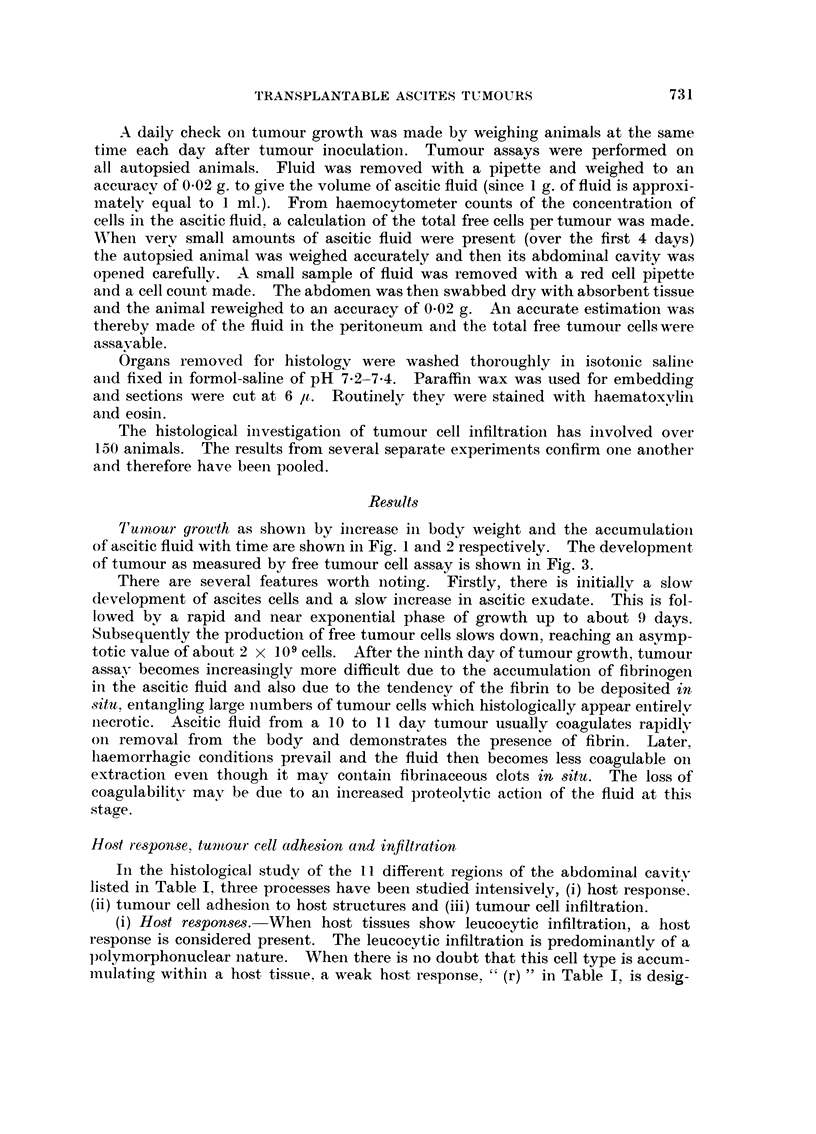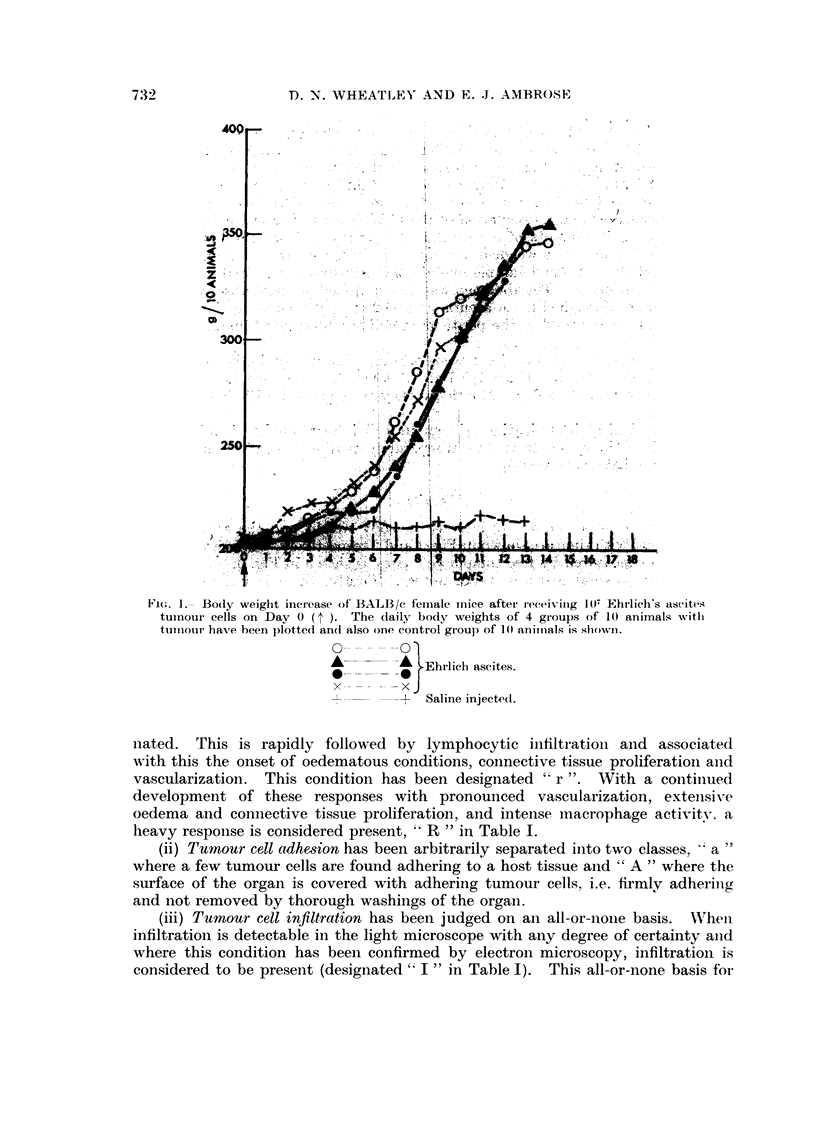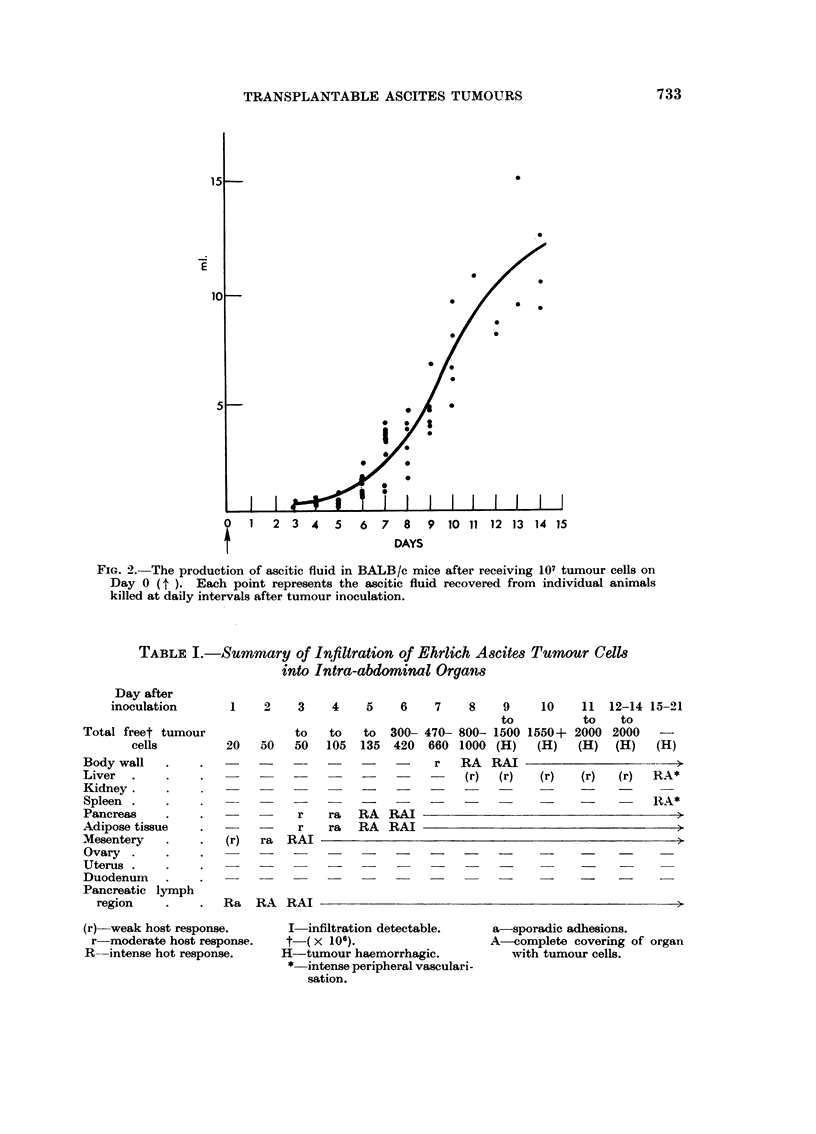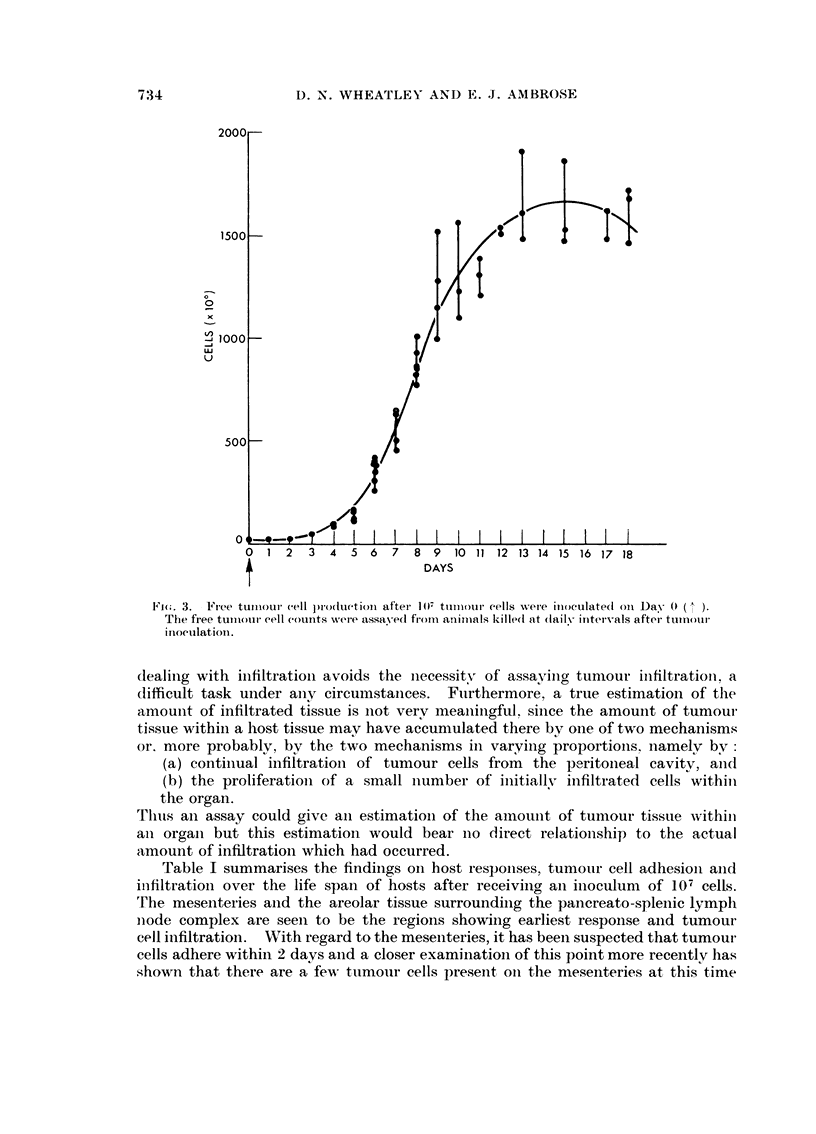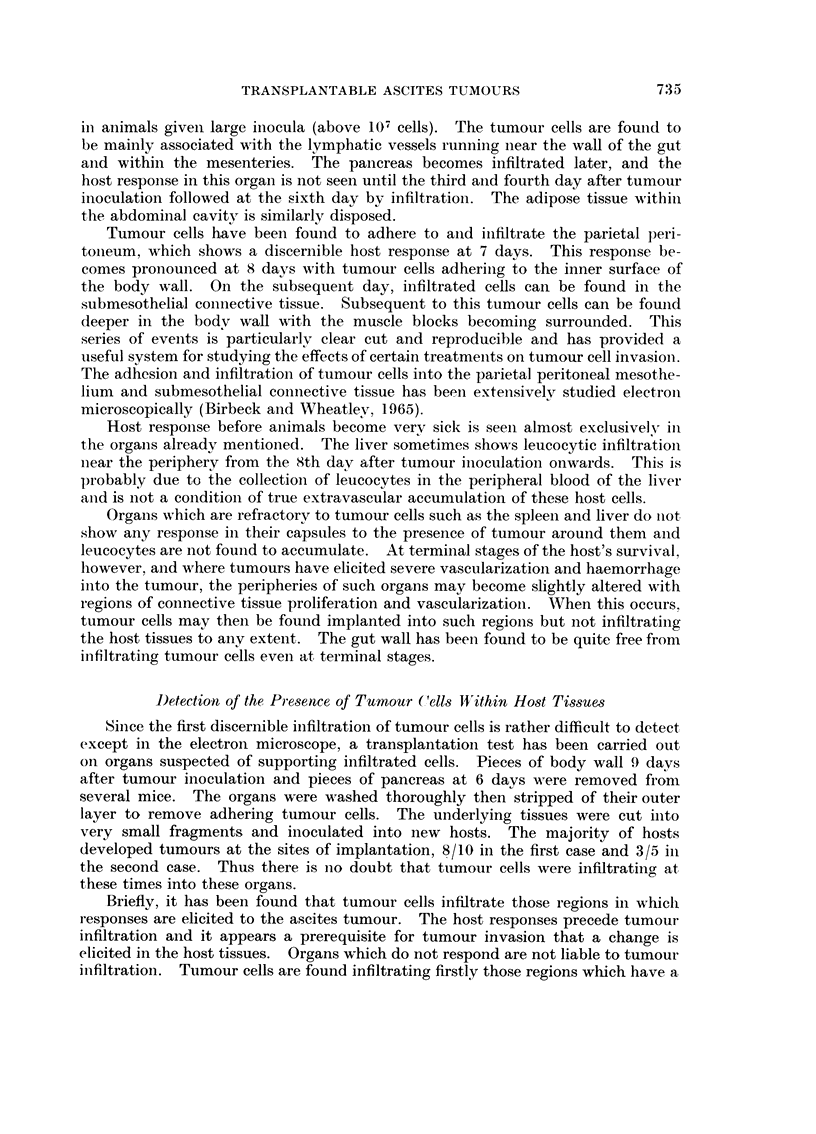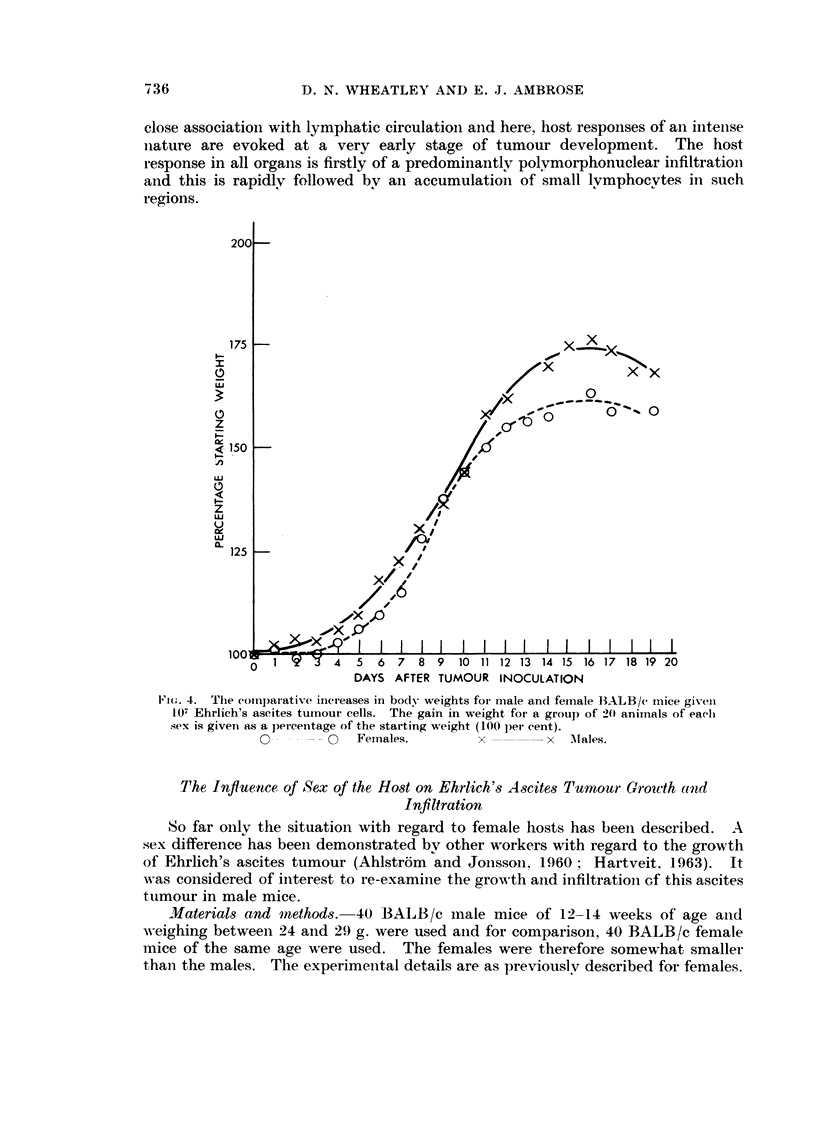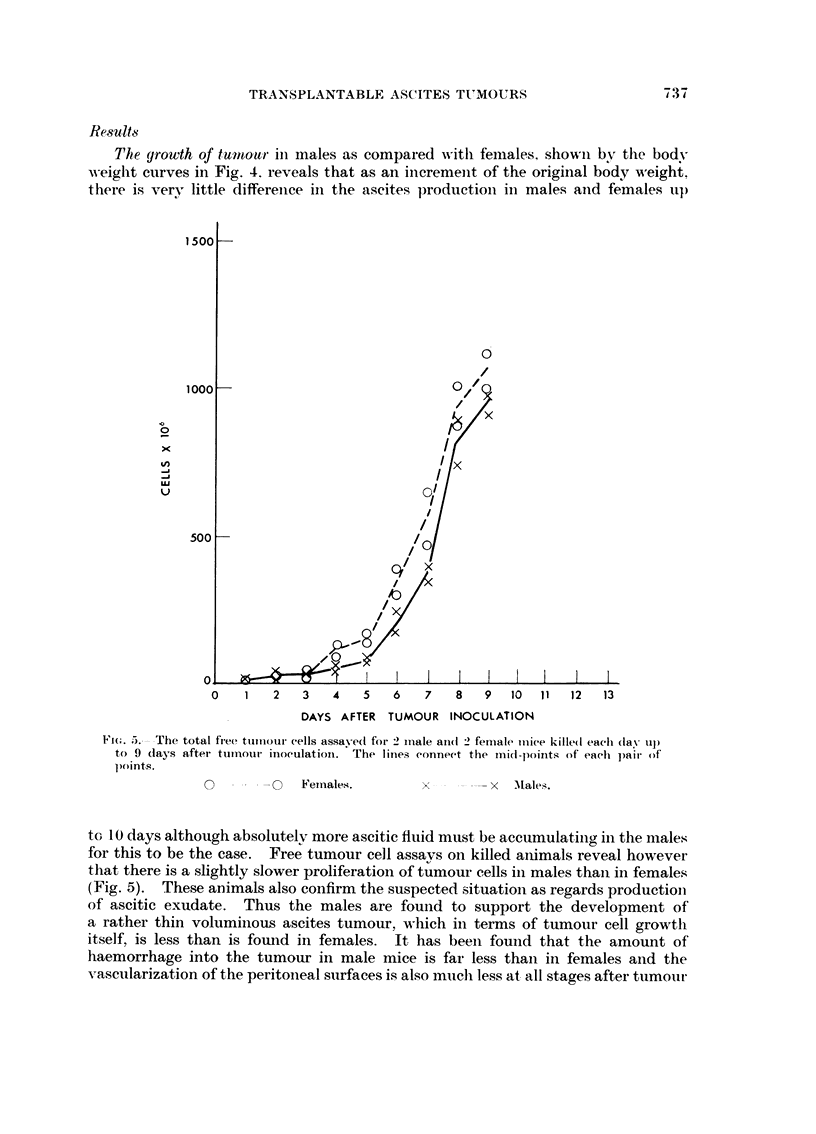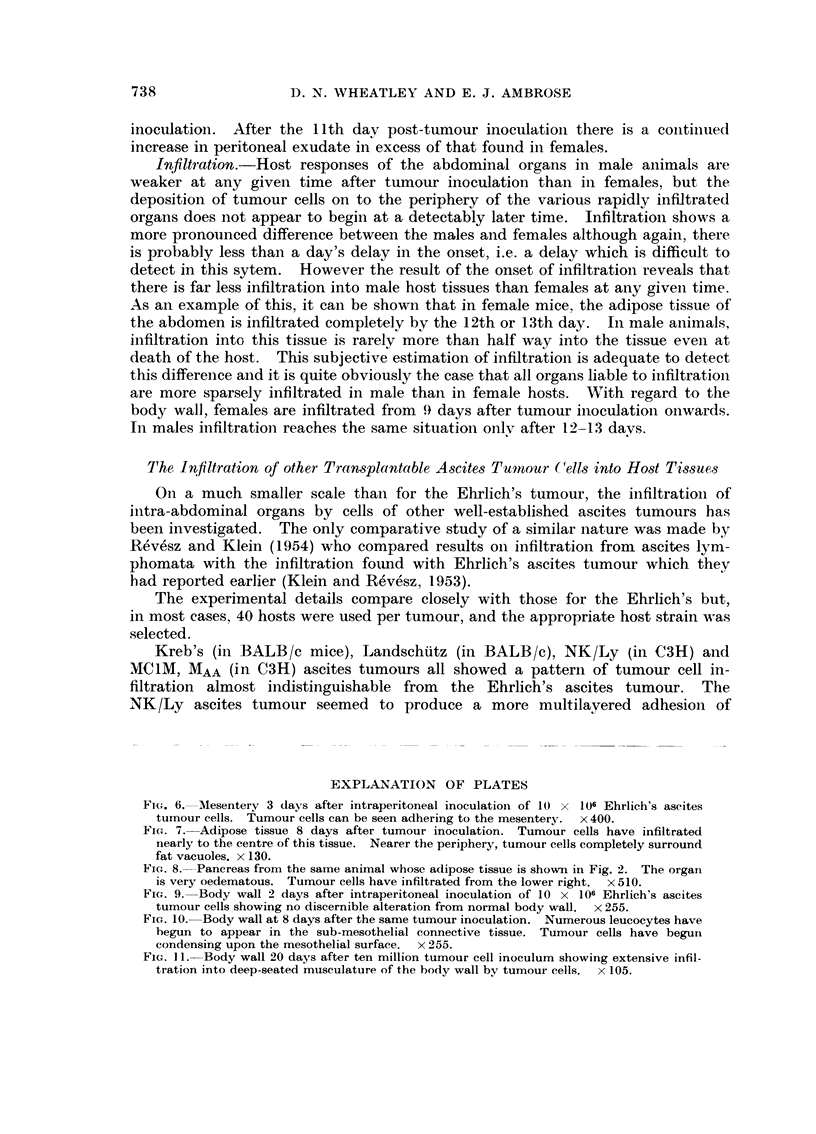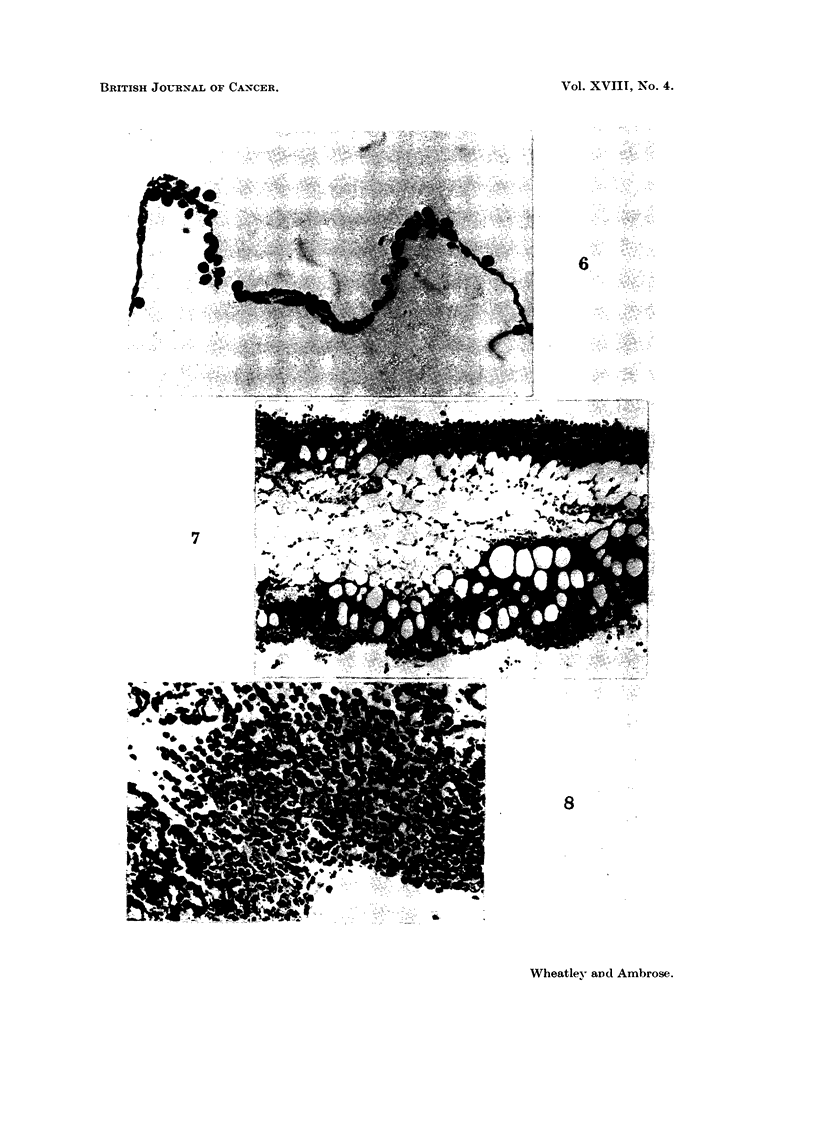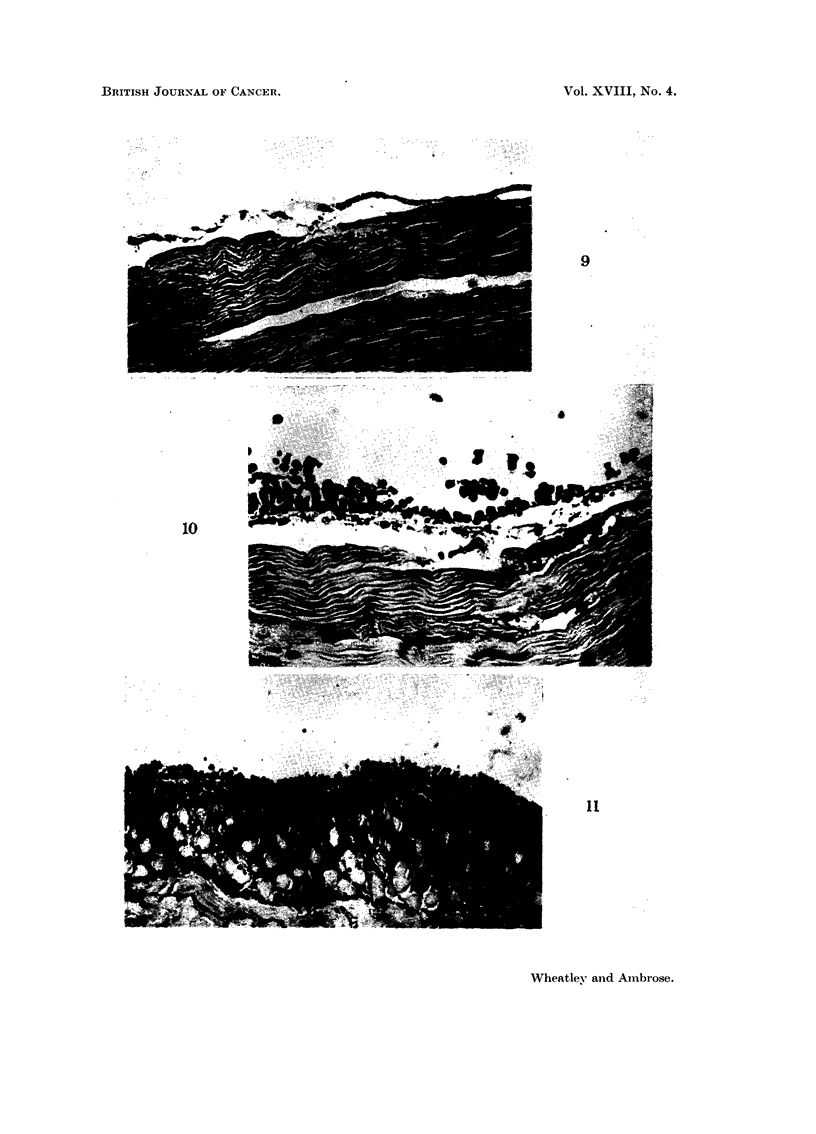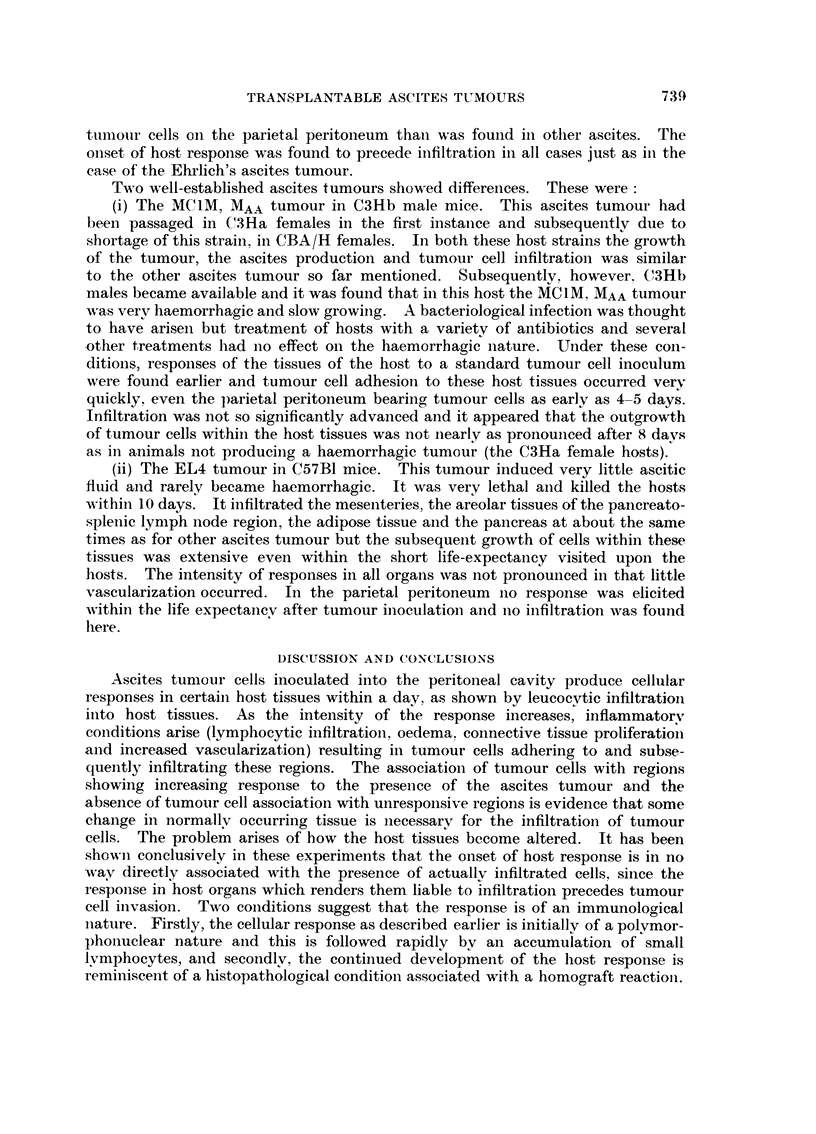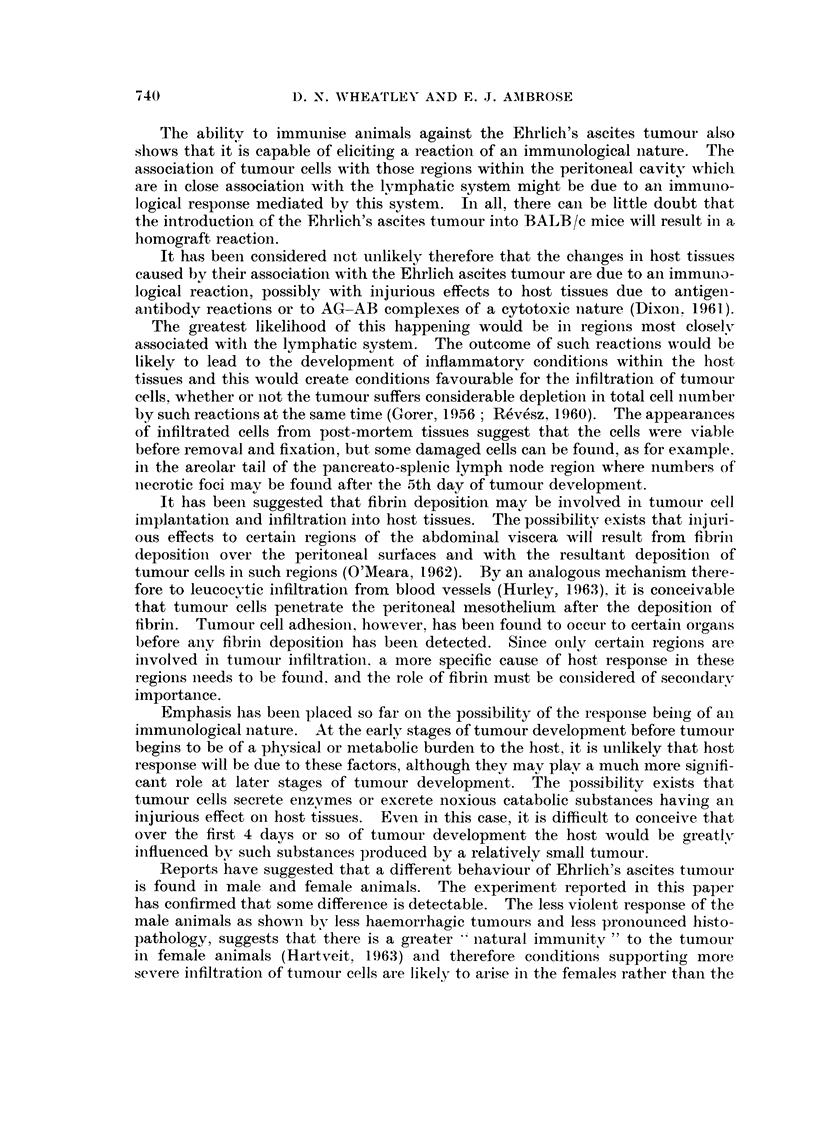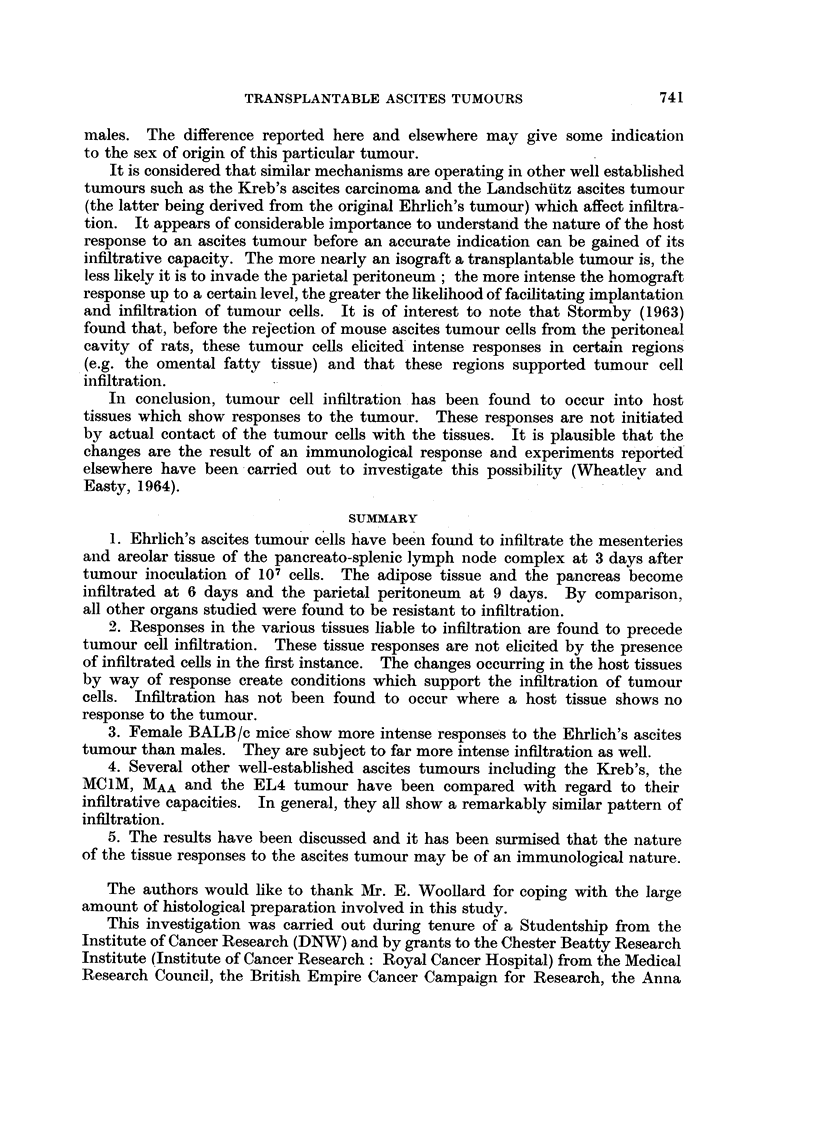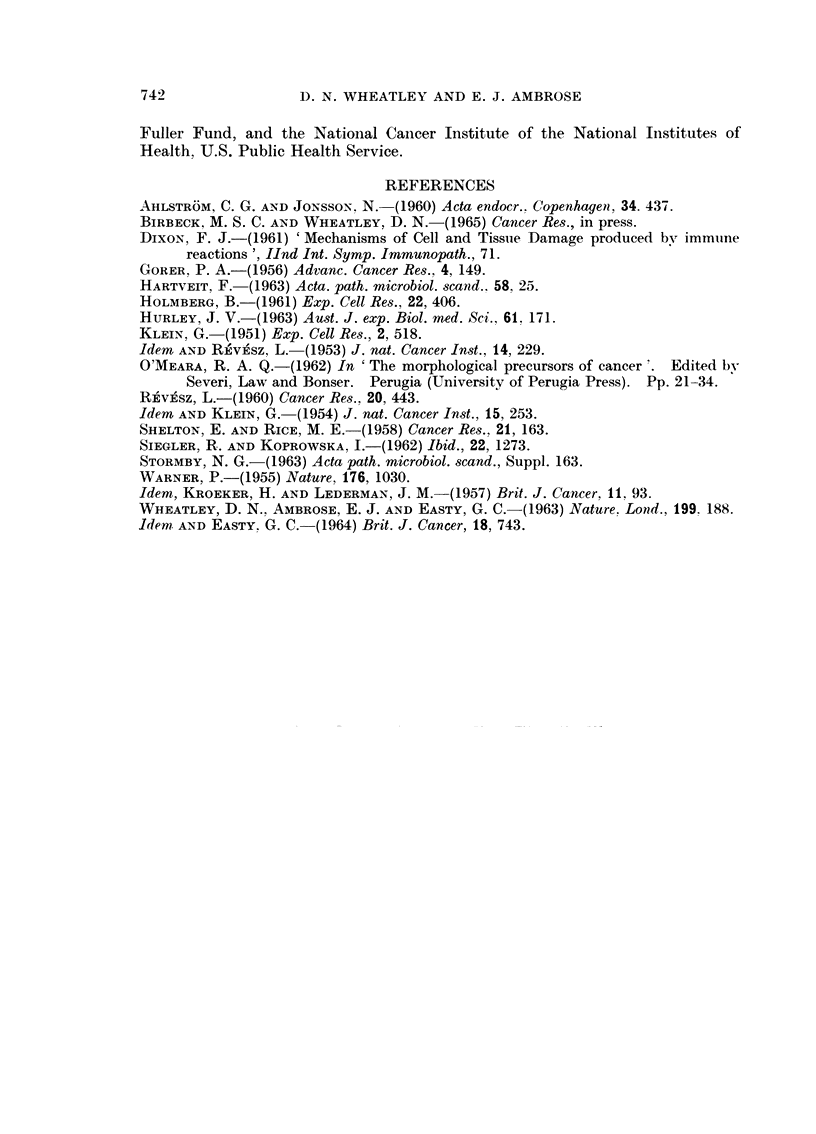# Tumour Cell Invasion from Transplantable Ascites Tumours into Host Tissues

**DOI:** 10.1038/bjc.1964.85

**Published:** 1964-12

**Authors:** D. N. Wheatley, E. J. Ambrose

## Abstract

**Images:**


### TUMOUR CELL INVASION FROM TRANSPLANTABLE ASCITES TUMOURS INTO HOST TISSUES

D. N. WHEATLEY* AND E. J. AMBROSE

*From the Chester Beatty Research Institute, Institute of Cancer Research : Royal Cancer Hospital, Fulham Road, London. S.W.3*

Received for publication June 29, 1964

THE infiltration of ascites tumour cells from ascitic fluid into the intra-abdominal organs of mice has been the subject of a brief report recently (Wheatley, Ambrose and Easty, 1963). This paper reports this work in full detail and also some associated work, thus giving a more complete coverage to the study of ascites tumour cell invasion.

Ascites tumour cells have been found to infiltrate certain organs within the abdominal cavity some time after tumour inoculation. Klein (1951) and Klein and Révész (1953) have found that the mesenteries, the pancreas and the adipose tissue are particularly prone to infiltration, becoming invaded between 4 to 5 days after tumour inoculation. Their findings have been corroborated by the results of other workers, e.g. Warner (1955) and Warner, Kroeker and Lederman, (1957), Shelton and Rice (1958), Siegler and Koprowska (1962) and Stormby (1963). A full investigation of the tissues of the abdominal cavity has not been attempted in any of these studies and the rate of tumour cell infiltration has only briefly been touched upon by Klein and Révész (1953) and Révész and Klein (1954) who estimated the time of onset of infiltration into the pancreas and omental fatty tissue. Since the processes involved in tumour cell infiltration and the distribution of tumour cells in the various host tissue have quite obvious bearings on tumour cell invasion and metastasis, a thorough investigation was undertaken.

### *Materials and Methods*

The major part of this work concerns the Ehrlich's ascites tumour of the near tetraploid strain, which was obtained from Klein in 1952. It has been passaged in BALB/c female mice and this strain has been used for most of the experiments reported here. Animals were 12–14 weeks of age and between 20 and 25 g. in weight when given tumour.

Tumour cells for inoculation were taken from animals bearing 8 day non-haemorrhagic ascites tumours. The concentration of tumour cells was adjusted with 0·85 per cent saline so that 0·2 ml. of fluid contained $10^7$ viable tumour cells. This was the standard inoculum. The viability of tumour cells was checked concomitantly with the haemocytometer counting of diluted ascitic fluid by a dye-exclusion test (0·5 per cent lissamine green ; Holmberg, 1961). Tumour cells were injected intraperitoneally by means of a No. 18 b.s.g. needle introduced through the lower right abdominal wall of the mouse.

* Present address : Department of Pathology, University Medical Buildings, Foresterhill, Aberdeen.

A daily check on tumour growth was made by weighing animals at the same time each day after tumour inoculation. Tumour assays were performed on all autopsied animals. Fluid was removed with a pipette and weighed to an accuracy of 0·02 g. to give the volume of ascitic fluid (since 1 g. of fluid is approximately equal to 1 ml.). From haemocytometer counts of the concentration of cells in the ascitic fluid, a calculation of the total free cells per tumour was made. When very small amounts of ascitic fluid were present (over the first 4 days) the autopsied animal was weighed accurately and then its abdominal cavity was opened carefully. A small sample of fluid was removed with a red cell pipette and a cell count made. The abdomen was then swabbed dry with absorbent tissue and the animal reweighed to an accuracy of 0·02 g. An accurate estimation was thereby made of the fluid in the peritoneum and the total free tumour cells were assayable.

Organs removed for histology were washed thoroughly in isotonic saline and fixed in formol-saline of pH 7·2–7·4. Paraffin wax was used for embedding and sections were cut at 6 $\mu$. Routinely they were stained with haematoxylin and eosin.

The histological investigation of tumour cell infiltration has involved over 150 animals. The results from several separate experiments confirm one another and therefore have been pooled.

### *Results*

*Tumour growth* as shown by increase in body weight and the accumulation of ascitic fluid with time are shown in Fig. 1 and 2 respectively. The development of tumour as measured by free tumour cell assay is shown in Fig. 3.

There are several features worth noting. Firstly, there is initially a slow development of ascites cells and a slow increase in ascitic exudate. This is followed by a rapid and near exponential phase of growth up to about 9 days. Subsequently the production of free tumour cells slows down, reaching an asymptotic value of about $2 \times 10^9$ cells. After the ninth day of tumour growth, tumour assay becomes increasingly more difficult due to the accumulation of fibrinogen in the ascitic fluid and also due to the tendency of the fibrin to be deposited *in situ*, entangling large numbers of tumour cells which histologically appear entirely necrotic. Ascitic fluid from a 10 to 11 day tumour usually coagulates rapidly on removal from the body and demonstrates the presence of fibrin. Later, haemorrhagic conditions prevail and the fluid then becomes less coagulable on extraction even though it may contain fibrinaceous clots *in situ*. The loss of coagulability may be due to an increased proteolytic action of the fluid at this stage.

#### *Host response, tumour cell adhesion and infiltration*

In the histological study of the 11 different regions of the abdominal cavity listed in Table I, three processes have been studied intensively, (i) host response, (ii) tumour cell adhesion to host structures and (iii) tumour cell infiltration.

(i) *Host responses.*—When host tissues show leucocytic infiltration, a host response is considered present. The leucocytic infiltration is predominantly of a polymorphonuclear nature. When there is no doubt that this cell type is accummulating within a host tissue, a weak host response, " (r) " in Table I, is desig-

FIG. 1.—Body weight increase of BALB/c female mice after receiving $10^7$ Ehrlich's ascites tumour cells on Day 0 (↑). The daily body weights of 4 groups of 10 animals with tumour have been plotted and also one control group of 10 animals is shown.

○– – – – –○, ▲———▲, ●———●, ×– – – – –× : Ehrlich ascites.

+——— ———+ : Saline injected.

nated. This is rapidly followed by lymphocytic infiltration and associated with this the onset of oedematous conditions, connective tissue proliferation and vascularization. This condition has been designated " r ". With a continued development of these responses with pronounced vascularization, extensive oedema and connective tissue proliferation, and intense macrophage activity, a heavy response is considered present, " R " in Table I.

(ii) *Tumour cell adhesion* has been arbitrarily separated into two classes, " a " where a few tumour cells are found adhering to a host tissue and " A " where the surface of the organ is covered with adhering tumour cells, i.e. firmly adhering and not removed by thorough washings of the organ.

(iii) *Tumour cell infiltration* has been judged on an all-or-none basis. When infiltration is detectable in the light microscope with any degree of certainty and where this condition has been confirmed by electron microscopy, infiltration is considered to be present (designated " I " in Table I). This all-or-none basis for

FIG. 2.—The production of ascitic fluid in BALB/c mice after receiving $10^7$ tumour cells on Day 0 (↑). Each point represents the ascitic fluid recovered from individual animals killed at daily intervals after tumour inoculation.

TABLE I.—*Summary of Infiltration of Ehrlich Ascites Tumour Cells into Intra-abdominal Organs*

| Day after inoculation | 1 | 2 | 3 | 4 | 5 | 6 | 7 | 8 | 9 | 10 | 11 | 12–14 | 15–21 |
|---|---|---|---|---|---|---|---|---|---|---|---|---|---|
| Total free† tumour cells | 20 | 50 | to 50 | to 105 | to 135 | 300–420 | 470–660 | 800–1000 | to 1500 (H) | 1550+ (H) | to 2000 (H) | to 2000 (H) | — (H) |
| Body wall . . | — | — | — | — | — | — | r | RA | RAI → | | | | → |
| Liver . . . | — | — | — | — | — | — | — | (r) | (r) | (r) | (r) | (r) | RA* |
| Kidney . . . | — | — | — | — | — | — | — | — | — | — | — | — | — |
| Spleen . . . | — | — | — | — | — | — | — | — | — | — | — | — | RA* |
| Pancreas . . | — | — | r | ra | RA | RAI → | | | | | | | → |
| Adipose tissue . | — | — | r | ra | RA | RAI → | | | | | | | → |
| Mesentery . . | (r) | ra | RAI → | | | | | | | | | | → |
| Ovary . . . | — | — | — | — | — | — | — | — | — | — | — | — | — |
| Uterus . . . | — | — | — | — | — | — | — | — | — | — | — | — | — |
| Duodenum . . | — | — | — | — | — | — | — | — | — | — | — | — | — |
| Pancreatic lymph region . . | Ra | RA | RAI → | | | | | | | | | | → |

(r)—weak host response.
r—moderate host response.
R—intense hot response.

I—infiltration detectable.
†—(× $10^6$).
H—tumour haemorrhagic.
*—intense peripheral vascularisation.

a—sporadic adhesions.
A—complete covering of organ with tumour cells.

FIG. 3. Free tumour cell production after $10^7$ tumour cells were inoculated on Day 0 (↑). The free tumour cell counts were assayed from animals killed at daily intervals after tumour inoculation.

dealing with infiltration avoids the necessity of assaying tumour infiltration, a difficult task under any circumstances. Furthermore, a true estimation of the amount of infiltrated tissue is not very meaningful, since the amount of tumour tissue within a host tissue may have accumulated there by one of two mechanisms or, more probably, by the two mechanisms in varying proportions, namely by :

(a) continual infiltration of tumour cells from the peritoneal cavity, and

(b) the proliferation of a small number of initially infiltrated cells within the organ.

Thus an assay could give an estimation of the amount of tumour tissue within an organ but this estimation would bear no direct relationship to the actual amount of infiltration which had occurred.

Table I summarises the findings on host responses, tumour cell adhesion and infiltration over the life span of hosts after receiving an inoculum of $10^7$ cells. The mesenteries and the areolar tissue surrounding the pancreato-splenic lymph node complex are seen to be the regions showing earliest response and tumour cell infiltration. With regard to the mesenteries, it has been suspected that tumour cells adhere within 2 days and a closer examination of this point more recently has shown that there are a few tumour cells present on the mesenteries at this time in animals given large inocula (above $10^7$ cells). The tumour cells are found to be mainly associated with the lymphatic vessels running near the wall of the gut and within the mesenteries. The pancreas becomes infiltrated later, and the host response in this organ is not seen until the third and fourth day after tumour inoculation followed at the sixth day by infiltration. The adipose tissue within the abdominal cavity is similarly disposed.

Tumour cells have been found to adhere to and infiltrate the parietal peritoneum, which shows a discernible host response at 7 days. This response becomes pronounced at 8 days with tumour cells adhering to the inner surface of the body wall. On the subsequent day, infiltrated cells can be found in the submesothelial connective tissue. Subsequent to this tumour cells can be found deeper in the body wall with the muscle blocks becoming surrounded. This series of events is particularly clear cut and reproducible and has provided a useful system for studying the effects of certain treatments on tumour cell invasion. The adhesion and infiltration of tumour cells into the parietal peritoneal mesothelium and submesothelial connective tissue has been extensively studied electron microscopically (Birbeck and Wheatley, 1965).

Host response before animals become very sick is seen almost exclusively in the organs already mentioned. The liver sometimes shows leucocytic infiltration near the periphery from the 8th day after tumour inoculation onwards. This is probably due to the collection of leucocytes in the peripheral blood of the liver and is not a condition of true extravascular accumulation of these host cells.

Organs which are refractory to tumour cells such as the spleen and liver do not show any response in their capsules to the presence of tumour around them and leucocytes are not found to accumulate. At terminal stages of the host's survival, however, and where tumours have elicited severe vascularization and haemorrhage into the tumour, the peripheries of such organs may become slightly altered with regions of connective tissue proliferation and vascularization. When this occurs, tumour cells may then be found implanted into such regions but not infiltrating the host tissues to any extent. The gut wall has been found to be quite free from infiltrating tumour cells even at terminal stages.

### *Detection of the Presence of Tumour Cells Within Host Tissues*

Since the first discernible infiltration of tumour cells is rather difficult to detect except in the electron microscope, a transplantation test has been carried out on organs suspected of supporting infiltrated cells. Pieces of body wall 9 days after tumour inoculation and pieces of pancreas at 6 days were removed from several mice. The organs were washed thoroughly then stripped of their outer layer to remove adhering tumour cells. The underlying tissues were cut into very small fragments and inoculated into new hosts. The majority of hosts developed tumours at the sites of implantation, 8/10 in the first case and 3/5 in the second case. Thus there is no doubt that tumour cells were infiltrating at these times into these organs.

Briefly, it has been found that tumour cells infiltrate those regions in which responses are elicited to the ascites tumour. The host responses precede tumour infiltration and it appears a prerequisite for tumour invasion that a change is elicited in the host tissues. Organs which do not respond are not liable to tumour infiltration. Tumour cells are found infiltrating firstly those regions which have a close association with lymphatic circulation and here, host responses of an intense nature are evoked at a very early stage of tumour development. The host response in all organs is firstly of a predominantly polymorphonuclear infiltration and this is rapidly followed by an accumulation of small lymphocytes in such regions.

FIG. 4. The comparative increases in body weights for male and female BALB/c mice given $10^7$ Ehrlich's ascites tumour cells. The gain in weight for a group of 20 animals of each sex is given as a percentage of the starting weight (100 per cent).

O- - - - O Females. X———X Males.

*The Influence of Sex of the Host on Ehrlich's Ascites Tumour Growth and Infiltration*

So far only the situation with regard to female hosts has been described. A sex difference has been demonstrated by other workers with regard to the growth of Ehrlich's ascites tumour (Ahlström and Jonsson, 1960; Hartveit, 1963). It was considered of interest to re-examine the growth and infiltration of this ascites tumour in male mice.

*Materials and methods.*—40 BALB/c male mice of 12–14 weeks of age and weighing between 24 and 29 g. were used and for comparison, 40 BALB/c female mice of the same age were used. The females were therefore somewhat smaller than the males. The experimental details are as previously described for females.

*Results*

*The growth of tumour* in males as compared with females, shown by the body weight curves in Fig. 4, reveals that as an increment of the original body weight, there is very little difference in the ascites production in males and females up

FIG. 5.—The total free tumour cells assayed for 2 male and 2 female mice killed each day up to 9 days after tumour inoculation. The lines connect the mid-points of each pair of points.

○ - - - ○ Females. × ——— × Males.

to 10 days although absolutely more ascitic fluid must be accumulating in the males for this to be the case. Free tumour cell assays on killed animals reveal however that there is a slightly slower proliferation of tumour cells in males than in females (Fig. 5). These animals also confirm the suspected situation as regards production of ascitic exudate. Thus the males are found to support the development of a rather thin voluminous ascites tumour, which in terms of tumour cell growth itself, is less than is found in females. It has been found that the amount of haemorrhage into the tumour in male mice is far less than in females and the vascularization of the peritoneal surfaces is also much less at all stages after tumour inoculation. After the 11th day post-tumour inoculation there is a continued increase in peritoneal exudate in excess of that found in females.

*Infiltration.*—Host responses of the abdominal organs in male animals are weaker at any given time after tumour inoculation than in females, but the deposition of tumour cells on to the periphery of the various rapidly infiltrated organs does not appear to begin at a detectably later time. Infiltration shows a more pronounced difference between the males and females although again, there is probably less than a day's delay in the onset, i.e. a delay which is difficult to detect in this sytem. However the result of the onset of infiltration reveals that there is far less infiltration into male host tissues than females at any given time. As an example of this, it can be shown that in female mice, the adipose tissue of the abdomen is infiltrated completely by the 12th or 13th day. In male animals, infiltration into this tissue is rarely more than half way into the tissue even at death of the host. This subjective estimation of infiltration is adequate to detect this difference and it is quite obviously the case that all organs liable to infiltration are more sparsely infiltrated in male than in female hosts. With regard to the body wall, females are infiltrated from 9 days after tumour inoculation onwards. In males infiltration reaches the same situation only after 12–13 days.

### *The Infiltration of other Transplantable Ascites Tumour Cells into Host Tissues*

On a much smaller scale than for the Ehrlich's tumour, the infiltration of intra-abdominal organs by cells of other well-established ascites tumours has been investigated. The only comparative study of a similar nature was made by Révész and Klein (1954) who compared results on infiltration from ascites lymphomata with the infiltration found with Ehrlich's ascites tumour which they had reported earlier (Klein and Révész, 1953).

The experimental details compare closely with those for the Ehrlich's but, in most cases, 40 hosts were used per tumour, and the appropriate host strain was selected.

Kreb's (in BALB/c mice), Landschütz (in BALB/c), NK/Ly (in C3H) and MC1M, $M_{AA}$ (in C3H) ascites tumours all showed a pattern of tumour cell infiltration almost indistinguishable from the Ehrlich's ascites tumour. The NK/Ly ascites tumour seemed to produce a more multilayered adhesion of

### EXPLANATION OF PLATES

FIG. 6.—Mesentery 3 days after intraperitoneal inoculation of $10 \times 10^6$ Ehrlich's ascites tumour cells. Tumour cells can be seen adhering to the mesentery. ×400.

FIG. 7.—Adipose tissue 8 days after tumour inoculation. Tumour cells have infiltrated nearly to the centre of this tissue. Nearer the periphery, tumour cells completely surround fat vacuoles. ×130.

FIG. 8.—Pancreas from the same animal whose adipose tissue is shown in Fig. 2. The organ is very oedematous. Tumour cells have infiltrated from the lower right. ×510.

FIG. 9.—Body wall 2 days after intraperitoneal inoculation of $10 \times 10^6$ Ehrlich's ascites tumour cells showing no discernible alteration from normal body wall. ×255.

FIG. 10.—Body wall at 8 days after the same tumour inoculation. Numerous leucocytes have begun to appear in the sub-mesothelial connective tissue. Tumour cells have begun condensing upon the mesothelial surface. ×255.

FIG. 11.—Body wall 20 days after ten million tumour cell inoculum showing extensive infiltration into deep-seated musculature of the body wall by tumour cells. ×105.

6

7

8

9

10

11

Wheatley and Ambrose.

tumour cells on the parietal peritoneum than was found in other ascites. The onset of host response was found to precede infiltration in all cases just as in the case of the Ehrlich's ascites tumour.

Two well-established ascites tumours showed differences. These were :

(i) The MC1M, $M_{AA}$ tumour in C3Hb male mice. This ascites tumour had been passaged in C3Ha females in the first instance and subsequently due to shortage of this strain, in CBA/H females. In both these host strains the growth of the tumour, the ascites production and tumour cell infiltration was similar to the other ascites tumour so far mentioned. Subsequently, however, C3Hb males became available and it was found that in this host the MC1M, $M_{AA}$ tumour was very haemorrhagic and slow growing. A bacteriological infection was thought to have arisen but treatment of hosts with a variety of antibiotics and several other treatments had no effect on the haemorrhagic nature. Under these conditions, responses of the tissues of the host to a standard tumour cell inoculum were found earlier and tumour cell adhesion to these host tissues occurred very quickly, even the parietal peritoneum bearing tumour cells as early as 4–5 days. Infiltration was not so significantly advanced and it appeared that the outgrowth of tumour cells within the host tissues was not nearly as pronounced after 8 days as in animals not producing a haemorrhagic tumour (the C3Ha female hosts).

(ii) The EL4 tumour in C57Bl mice. This tumour induced very little ascitic fluid and rarely became haemorrhagic. It was very lethal and killed the hosts within 10 days. It infiltrated the mesenteries, the areolar tissues of the pancreato-splenic lymph node region, the adipose tissue and the pancreas at about the same times as for other ascites tumour but the subsequent growth of cells within these tissues was extensive even within the short life-expectancy visited upon the hosts. The intensity of responses in all organs was not pronounced in that little vascularization occurred. In the parietal peritoneum no response was elicited within the life expectancy after tumour inoculation and no infiltration was found here.

### DISCUSSION AND CONCLUSIONS

Ascites tumour cells inoculated into the peritoneal cavity produce cellular responses in certain host tissues within a day, as shown by leucocytic infiltration into host tissues. As the intensity of the response increases, inflammatory conditions arise (lymphocytic infiltration, oedema, connective tissue proliferation and increased vascularization) resulting in tumour cells adhering to and subsequently infiltrating these regions. The association of tumour cells with regions showing increasing response to the presence of the ascites tumour and the absence of tumour cell association with unresponsive regions is evidence that some change in normally occurring tissue is necessary for the infiltration of tumour cells. The problem arises of how the host tissues become altered. It has been shown conclusively in these experiments that the onset of host response is in no way directly associated with the presence of actually infiltrated cells, since the response in host organs which renders them liable to infiltration precedes tumour cell invasion. Two conditions suggest that the response is of an immunological nature. Firstly, the cellular response as described earlier is initially of a polymorphonuclear nature and this is followed rapidly by an accumulation of small lymphocytes, and secondly, the continued development of the host response is reminiscent of a histopathological condition associated with a homograft reaction.

The ability to immunise animals against the Ehrlich's ascites tumour also shows that it is capable of eliciting a reaction of an immunological nature. The association of tumour cells with those regions within the peritoneal cavity which are in close association with the lymphatic system might be due to an immunological response mediated by this system. In all, there can be little doubt that the introduction of the Ehrlich's ascites tumour into BALB/c mice will result in a homograft reaction.

It has been considered not unlikely therefore that the changes in host tissues caused by their association with the Ehrlich ascites tumour are due to an immunological reaction, possibly with injurious effects to host tissues due to antigen-antibody reactions or to AG–AB complexes of a cytotoxic nature (Dixon, 1961). The greatest likelihood of this happening would be in regions most closely associated with the lymphatic system. The outcome of such reactions would be likely to lead to the development of inflammatory conditions within the host tissues and this would create conditions favourable for the infiltration of tumour cells, whether or not the tumour suffers considerable depletion in total cell number by such reactions at the same time (Gorer, 1956 ; Révész, 1960). The appearances of infiltrated cells from post-mortem tissues suggest that the cells were viable before removal and fixation, but some damaged cells can be found, as for example, in the areolar tail of the pancreato-splenic lymph node region where numbers of necrotic foci may be found after the 5th day of tumour development.

It has been suggested that fibrin deposition may be involved in tumour cell implantation and infiltration into host tissues. The possibility exists that injurious effects to certain regions of the abdominal viscera will result from fibrin deposition over the peritoneal surfaces and with the resultant deposition of tumour cells in such regions (O'Meara, 1962). By an analogous mechanism therefore to leucocytic infiltration from blood vessels (Hurley, 1963), it is conceivable that tumour cells penetrate the peritoneal mesothelium after the deposition of fibrin. Tumour cell adhesion, however, has been found to occur to certain organs before any fibrin deposition has been detected. Since only certain regions are involved in tumour infiltration, a more specific cause of host response in these regions needs to be found, and the role of fibrin must be considered of secondary importance.

Emphasis has been placed so far on the possibility of the response being of an immunological nature. At the early stages of tumour development before tumour begins to be of a physical or metabolic burden to the host, it is unlikely that host response will be due to these factors, although they may play a much more significant role at later stages of tumour development. The possibility exists that tumour cells secrete enzymes or excrete noxious catabolic substances having an injurious effect on host tissues. Even in this case, it is difficult to conceive that over the first 4 days or so of tumour development the host would be greatly influenced by such substances produced by a relatively small tumour.

Reports have suggested that a different behaviour of Ehrlich's ascites tumour is found in male and female animals. The experiment reported in this paper has confirmed that some difference is detectable. The less violent response of the male animals as shown by less haemorrhagic tumours and less pronounced histopathology, suggests that there is a greater " natural immunity " to the tumour in female animals (Hartveit, 1963) and therefore conditions supporting more severe infiltration of tumour cells are likely to arise in the females rather than the males. The difference reported here and elsewhere may give some indication to the sex of origin of this particular tumour.

It is considered that similar mechanisms are operating in other well established tumours such as the Kreb's ascites carcinoma and the Landschütz ascites tumour (the latter being derived from the original Ehrlich's tumour) which affect infiltration. It appears of considerable importance to understand the nature of the host response to an ascites tumour before an accurate indication can be gained of its infiltrative capacity. The more nearly an isograft a transplantable tumour is, the less likely it is to invade the parietal peritoneum ; the more intense the homograft response up to a certain level, the greater the likelihood of facilitating implantation and infiltration of tumour cells. It is of interest to note that Stormby (1963) found that, before the rejection of mouse ascites tumour cells from the peritoneal cavity of rats, these tumour cells elicited intense responses in certain regions (e.g. the omental fatty tissue) and that these regions supported tumour cell infiltration.

In conclusion, tumour cell infiltration has been found to occur into host tissues which show responses to the tumour. These responses are not initiated by actual contact of the tumour cells with the tissues. It is plausible that the changes are the result of an immunological response and experiments reported elsewhere have been carried out to investigate this possibility (Wheatley and Easty, 1964).

SUMMARY

1. Ehrlich's ascites tumour cells have been found to infiltrate the mesenteries and areolar tissue of the pancreato-splenic lymph node complex at 3 days after tumour inoculation of $10^7$ cells. The adipose tissue and the pancreas become infiltrated at 6 days and the parietal peritoneum at 9 days. By comparison, all other organs studied were found to be resistant to infiltration.

2. Responses in the various tissues liable to infiltration are found to precede tumour cell infiltration. These tissue responses are not elicited by the presence of infiltrated cells in the first instance. The changes occurring in the host tissues by way of response create conditions which support the infiltration of tumour cells. Infiltration has not been found to occur where a host tissue shows no response to the tumour.

3. Female BALB/c mice show more intense responses to the Ehrlich's ascites tumour than males. They are subject to far more intense infiltration as well.

4. Several other well-established ascites tumours including the Kreb's, the MC1M, $M_{AA}$ and the EL4 tumour have been compared with regard to their infiltrative capacities. In general, they all show a remarkably similar pattern of infiltration.

5. The results have been discussed and it has been surmised that the nature of the tissue responses to the ascites tumour may be of an immunological nature.

The authors would like to thank Mr. E. Woollard for coping with the large amount of histological preparation involved in this study.

This investigation was carried out during tenure of a Studentship from the Institute of Cancer Research (DNW) and by grants to the Chester Beatty Research Institute (Institute of Cancer Research : Royal Cancer Hospital) from the Medical Research Council, the British Empire Cancer Campaign for Research, the Anna

Fuller Fund, and the National Cancer Institute of the National Institutes of Health, U.S. Public Health Service.

### REFERENCES

AHLSTRÖM, C. G. AND JONSSON, N.—(1960) *Acta endocr., Copenhagen*, **34**. 437.
BIRBECK, M. S. C. AND WHEATLEY, D. N.—(1965) *Cancer Res.*, in press.
DIXON, F. J.—(1961) 'Mechanisms of Cell and Tissue Damage produced by immune reactions', *IInd Int. Symp. Immunopath.*, 71.
GORER, P. A.—(1956) *Advanc. Cancer Res.*, **4**, 149.
HARTVEIT, F.—(1963) *Acta. path. microbiol. scand.*, **58**, 25.
HOLMBERG, B.—(1961) *Exp. Cell Res.*, **22**, 406.
HURLEY, J. V.—(1963) *Aust. J. exp. Biol. med. Sci.*, **61**, 171.
KLEIN, G.—(1951) *Exp. Cell Res.*, **2**, 518.
*Idem* AND RÉVÉSZ, L.—(1953) *J. nat. Cancer Inst.*, **14**, 229.
O'MEARA, R. A. Q.—(1962) *In* 'The morphological precursors of cancer'. Edited by Severi, Law and Bonser. Perugia (University of Perugia Press). Pp. 21–34.
RÉVÉSZ, L.—(1960) *Cancer Res.*, **20**, 443.
*Idem* AND KLEIN, G.—(1954) *J. nat. Cancer Inst.*, **15**, 253.
SHELTON, E. AND RICE, M. E.—(1958) *Cancer Res.*, **21**, 163.
SIEGLER, R. AND KOPROWSKA, I.—(1962) *Ibid.*, **22**, 1273.
STORMBY, N. G.—(1963) *Acta path. microbiol. scand.*, Suppl. 163.
WARNER, P.—(1955) *Nature*, **176**, 1030.
*Idem*, KROEKER, H. AND LEDERMAN, J. M.—(1957) *Brit. J. Cancer*, **11**, 93.
WHEATLEY, D. N., AMBROSE, E. J. AND EASTY, G. C.—(1963) *Nature, Lond.*, **199**, 188.
*Idem* AND EASTY, G. C.—(1964) *Brit. J. Cancer*, **18**, 743.